# Hydrocarbons in phlogopite from Kasenyi kamafugitic rocks (SW Uganda): cross-correlated AFM, confocal microscopy and Raman imaging

**DOI:** 10.1038/srep40663

**Published:** 2017-01-18

**Authors:** Daniele Moro, Giovanni Valdrè, Ernesto Mesto, Fernando Scordari, Maria Lacalamita, Giancarlo Della Ventura, Fabio Bellatreccia, Salvatore Scirè, Emanuela Schingaro

**Affiliations:** 1 Università di Bologna, Dipartimento di Scienze Biologiche, Geologiche e Ambientali, Piazza di Porta S. Donato 1, 40126 Bologna, Italy; 2 Università degli Studi di Bari ”Aldo Moro”, Dipartimento di Scienze della Terra e Geoambientali, Via E. Orabona 4, 70125 Bari, Italy; 3 Università Roma Tre, Dipartimento di Scienze, Largo S. Leonardo Murialdo 1, 00146 Rome, Italy; 4 Università degli Studi di Catania, Dipartimento di Scienze Chimiche, Viale A. Doria 6, 95125 Catania, Italy

## Abstract

This study presents a cross-correlated surface and near surface investigation of two phlogopite polytypes from Kasenyi kamafugitic rocks (SW Uganda) by means of advanced Atomic Force Microscopy (AFM), confocal microscopy and Raman micro-spectroscopy. AFM revealed comparable nanomorphology and electrostatic surface potential for the two mica polytypes. A widespread presence of nano-protrusions located on the mica flake surface was also observed, with an aspect ratio (maximum height/maximum width) from 0.01 to 0.09. Confocal microscopy showed these features to range from few nm to several *μ*m in dimension, and shapes from perfectly circular to ellipsoidic and strongly elongated. Raman spectra collected across the bubbles showed an intense and convolute absorption in the range 3000–2800 cm^−1^, associated with weaker bands at 1655, 1438 and 1297 cm^−1^, indicating the presence of fluid inclusions consisting of aliphatic hydrocarbons, alkanes and cycloalkanes, with minor amounts of oxygenated compounds, such as carboxylic acids. High-resolution Raman images provided evidence that these hydrocarbons are confined within the bubbles. This work represents the first direct evidence that phlogopite, a common rock-forming mineral, may be a possible reservoir for hydrocarbons.

Hydrocarbon fluid inclusions (HCFI) occur in fractures and microcracks of rocks and veins within forming minerals, e.g. quartz, calcite and pyroxene, and may consist of hydrocarbon oil, H_2_O, gases such as CO_2_ and CH_4_ or solids entrapped along with/or precipitated from the liquid phase at later times^1^,^2^. Hydrocarbons may derive by biogenic (thermogenesis; bacteriogenesis) or abiogenic processes. The latter are generally based on the reduction of carbon dioxide during magma cooling or, in hydrothermal systems, during water-rock interactions leading, for example, to serpentinization of ultramafic rocks. Abiogenic hydrocarbons may contribute to economic hydrocarbon reservoirs^3^. However, to discriminate between abiogenic and biogenic hydrocarbons, careful C and H isotope analyses are required^4^. The analysis of the HCFI may be performed on the bulk rock by crushing of the sample or on single inclusions by means of micro-spectroscopy (e.g. optical, infrared, Raman and fluorescence spectroscopy). To the best of the authors knowledge, HCFI have never been reported in phlogopite. The bulk properties of this common rock-forming mineral have been widely investigated, whereas little is known on its surface properties. In the present study, we relate on the nanoscale investigation of the surface to sub-surface of two phlogopite 1*M* and 2*M*_1_ polytypes, coexisting in a ultrapotassic kamafugite from Kasenyi together to a rare 3*T* polytype (south west Uganda, west branch of the East African Rift, see refs 5, 6). The first polytype has the structural formula: (K_0.91_Na_0.05_Ba_0.01_) (Mg_2.33_Al_0.08_
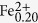

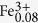
Ti_0.26_Cr_0.05_Ni_0.01_) (Si_2.87_Al_1.06_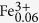)O_10.58_F_0.11_OH_1.31_. The other polytypes show substantially the same crystal-chemical formula with a greater extent of oxy-substitutions^5^,^6^. The observation of widespread nano- to micron-sized bubbles on the mica flakes prompted this multidisciplinary study, carried out by combining AFM, confocal microscopy, and Raman imaging.

## Results

Atomic force microscopy (AFM) is a well established methodology for the study of nanomorphologies and surface properties of layer silicates at the nanometer scale^7^,^8^,^9^. Recently, the AFM was used to investigate the *in situ* K^+^ − Na^+^ ion exchange in phlogopite single-crystals^10^. Uniform and non uniform swelling of the interlayer distance at the resolution of a single layer was revealed.

In the present study, a single crystal of 1*M*-phlogopite, labelled BU1–7, and two crystals of 2*M*_1_-phlogopite (BU1–15 and BU1–17, respectively^5^), were selected as representative of the samples population to investigate their surface properties at the nanoscale. The selected phlogopites were prepared for the AFM observations by fixing them onto AFM specimen mounting metal discs with double-sided adhesive tape. A fresh surface was obtained by adhesive tape-based cleavage.

### Morphology of cleavage edges and steps

**Figure 1:**
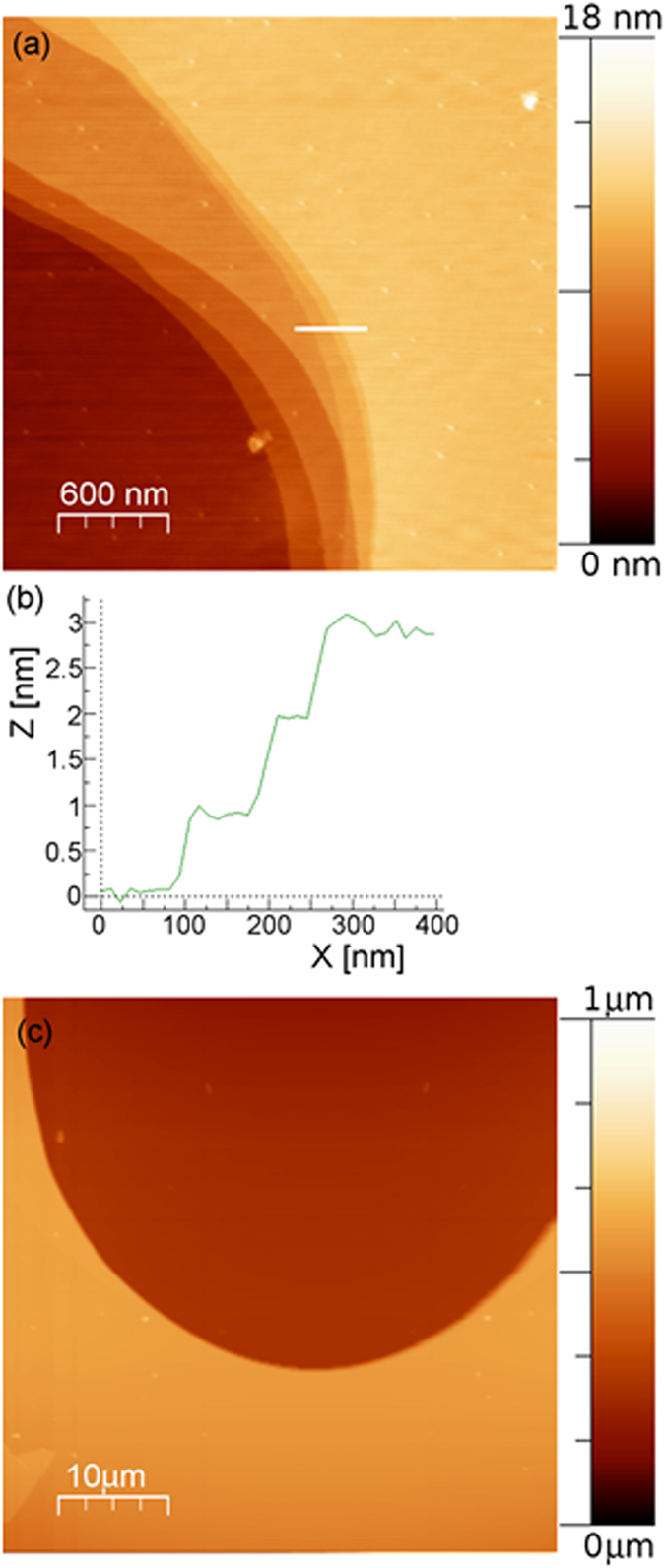
(**a**) AFM topographic image of BU1-7 phlogopite (001) surface with single T-O-T layer cleavage planes presenting a high radius of curvature. (**b**) Topographic profile measured along the white line section in (**a**). Three steps of about 1 nm corresponding to single T-O-T layers are measured. (**c**) AFM topographic image of a freshly cleaved phlogopite surface revealing a conchoidal cleavage.

AFM analysis showed, for both polytypes, similar morphological features in terms of cleavage edges, surface steps, roughness, AFM phase signal variation and surface potential. Phlogopite (001) surface was observed to present atomically flat domains uninterruptedly extended for more than 2500 *μ*m^2^. RMS roughness analysis gave values under the z position noise of the piezoelectric scanner (0.1 nm). The extension of the atomically flat domains is limited by cleavage edges that generate surface steps typically less than about 50 nm in height, and not exceeding about 400 nm in the investigated areas. Surface steps of about 1 nm were also measured corresponding to a single T-O-T layer (Fig. 1).

In general, the edge profiles were never jagged, but occurred mainly as perfectly straight lines, extending for tens of micrometres, or slightly curved ones. Furthermore, highly curved edges were also observed, with radius of curvature of less than 2 *μ*m (Fig. 1). Figure 1a displays a 3 *μ*m × 3 *μ*m AFM topographic image of terraces and curved cleavage features at the (001) phlogopite surface. Figure 1b shows a topographic profile measured along the white line section drawn in Fig. 1a, where three steps of about 1 nm, corresponding to single T-O-T layers, separate structurally flat domains. Figure 1c shows a cleavage feature that suggests a conchoidal form of cleavage not related to a crystallographic direction.

**Figure 2:**
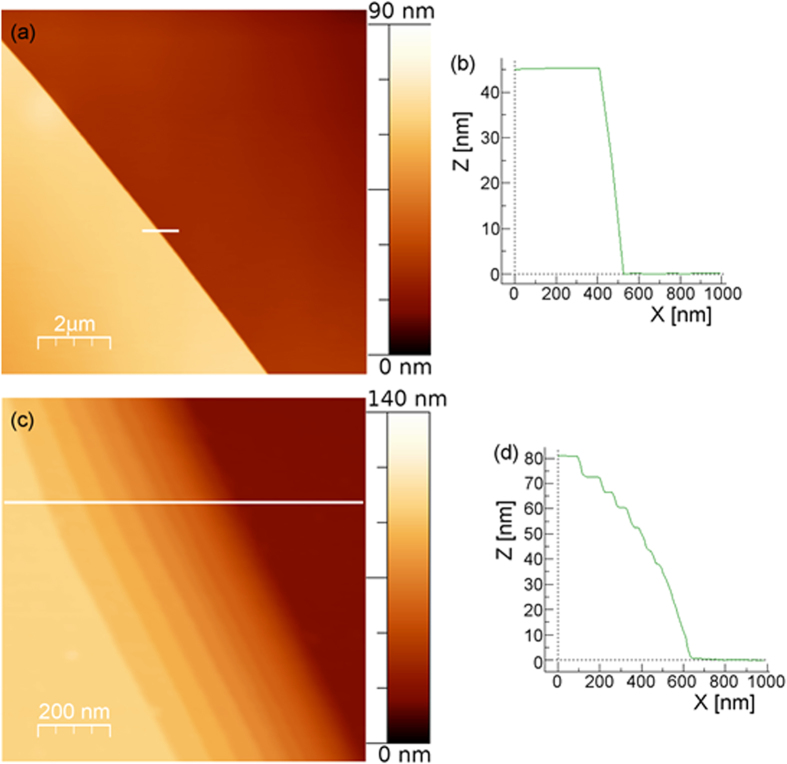
(**a**) AFM topographic image and (**b**) section profile of a single-step ledge of BU1-7. (**c**) AFM topographic image and (**d**) section profile of a stepped ledge for the same crystal.

Multilayer steps, observed with our AFM set up and tips, appeared as sharp single-step ledges or as stepped ledges (Fig. 2). Figure 2a shows, as an example, an AFM topographic image of a linear edge profile that separates two atomically flat terraces extended for several micrometres. The section profile (Fig. 2b) appears as a single-step ledge of about 45 nm. Figure 2c and d show, respectively, an AFM topographic image and a section profile of a stepped ledge of about 80 nm presenting sub-steps less than 9 nm in height.

The morphological investigation was complemented with phase signal analysis, while imaging was obtained in intermittent contact mode, and Kelvin probe measurements. The phase signal is the phase lag between the excitation of the cantilever and the cantilever response perturbed by the tip-to-sample interaction. It is qualitatively related to various material-based surface properties at the nanometre scale such as, composition variations (e.g., inclusions), visco-elasticity, adhesion, friction and surface charge. For the investigated areas of both polytypes the phase signal analysis revealed homogeneity and isotropy of the (001) surface properties. Kelvin probe force microscopy measurements gave a negative electric surface potential of the order of hundreds of mV, featuring no significant variations between the two polytypes.

### Morphology of nano-protrusions

Widespread nano-protrusions were observed on the surface of BU1–7, of BU1–15 in smaller amounts, and none on the surface of BU1–17 sample. The lateral extension of the nano-protrusions was typically of the order of hundreds of nanometers to several micrometers and the height from few nanometers to tens of nanometers. Generally, an aspect ratio (maximum height/maximum width) from 0.01 to 0.09 was measured and the shape of the projection onto the (001) crystallographic plane of phlogopite was mostly circular or elliptic.

On the surface of sample BU1–15 the nano-protrusions presented mostly (greater than 70%) a circular shape of the projection on the (001) plane, the remainders having an elliptical shape. Circular shaped nano-protrusions had a diameter between about 250 nm and 1900 nm, whereas the elliptical nano-protrusions featured higher lateral dimensions, between about 1 *μ*m and 4 *μ*m. The height typically ranged between about 2 nm and 40 nm, reaching 100 nm for the elliptical nano-protrusions. Sample BU1–7 featured a higher variability in the morphology of the nano-protrusions. Both circular and elliptic shapes were observed, and groups of clustered nano-protrusions and highly elongated structures were present.

**Figure 3:**
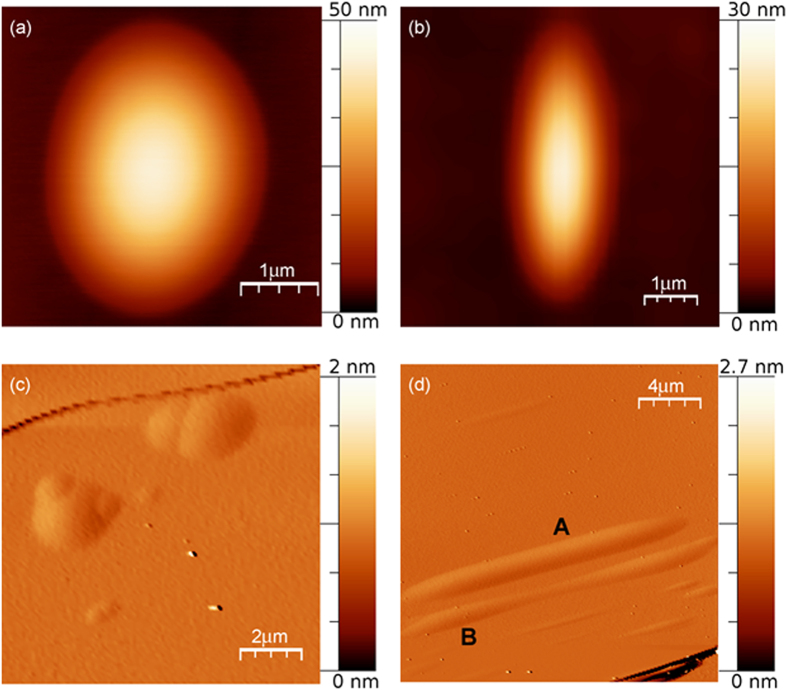
BU1-7 crystal: (**a**) and (**b**) AFM topographic images of surface nano-protrusions of pseudo-circular and elliptical shape, respectively. (**c**) Groups of clustered nano-protrusions. (**d**) Elongated-type nano-protrusions following a cleavage plane.

Figure 3 shows, as an example, some AFM images of nano-protrusions of the phlogopite surface with different shapes. The nano-protrusion of Fig. 3a presents a pseudo-circular contrast with a diameter of about 3.5 *μ*m and a height of about 40 nm, making it morphologically detectable only by atomic force microscopy. Figure 3b shows a protrusion with an elliptical geometry with a long axis of about 5.5 *μ*m, a short axis of about 2.3 *μ*m and a height of about 25 nm. Protrusions in Fig. 3c and d have more complex shapes. To better highlight and visualize the edges of these nano-protrusions, the images obtained from the amplitude signal are given. The amplitude signal is related to the error of the feedback control system in maintaining an imposed constant oscillation amplitude during the raster scan. Some of these surface features can be described from a geometrical point of view as intersections of the pseudo-circular nano-protrusions previously described (Fig. 3); other nano-protrusions can be described as elliptical, with a high long axis/short axis ratio (Fig. 3, labelled A) and as elongated nano-protrusions with a variable width (Fig. 3, labelled B). Figure 3 shows several elongated morphologies, whose orientation follows a cleavage edge (in the lower right corner).

**Figure 4:**
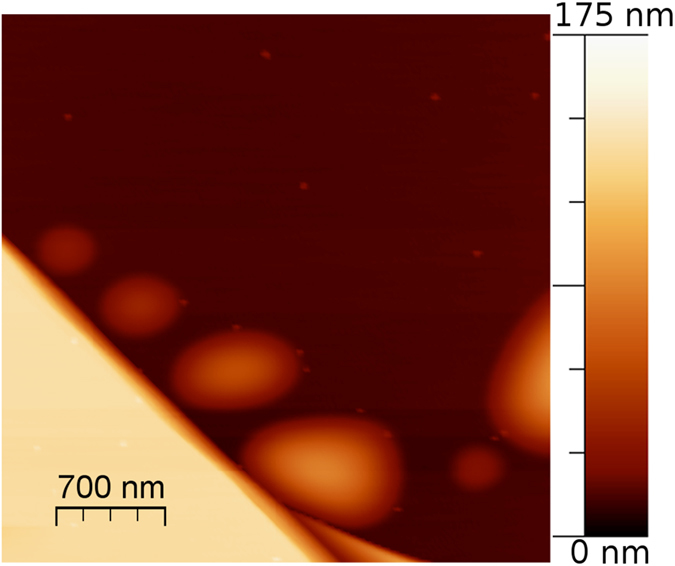
AFM topographic image of a sequence of nanoprotrusions with linearly increasing diameter and height, following a cleavage edge on BU1-7.

Nano-protrusions of different shape were observed to be aligned or elongated in a direction parallel to cleavage edges (Figs 3d and 4). Figure 4 presents, as an example, an AFM topographic image of a sequence of pseudo-circular features aligned along a cleavage edge. The sequence has a roughly linear increment of the diameters, from 400 nm to 1100 nm, and heights, from 18 nm to 78 nm. Similar sequences of nano-protrusions, with an approximately linear increment of their diameter, were also observed distant from the cleavage edges.

The analysis of the phase signal contrast did not provide qualitative evidence of a variation of the surface viscoelastic properties on the nano-protrusions with respect to the flat domains. No surface potential variation was detected. This suggests that the nano-protrusions of the surface are the effect of a sub-surface cause. Further investigations were devised employing confocal microscopy and Raman spectroscopy to assess the nature of the nano-protrusions.

### Chemical nature of the inclusions

**Figure 5:**
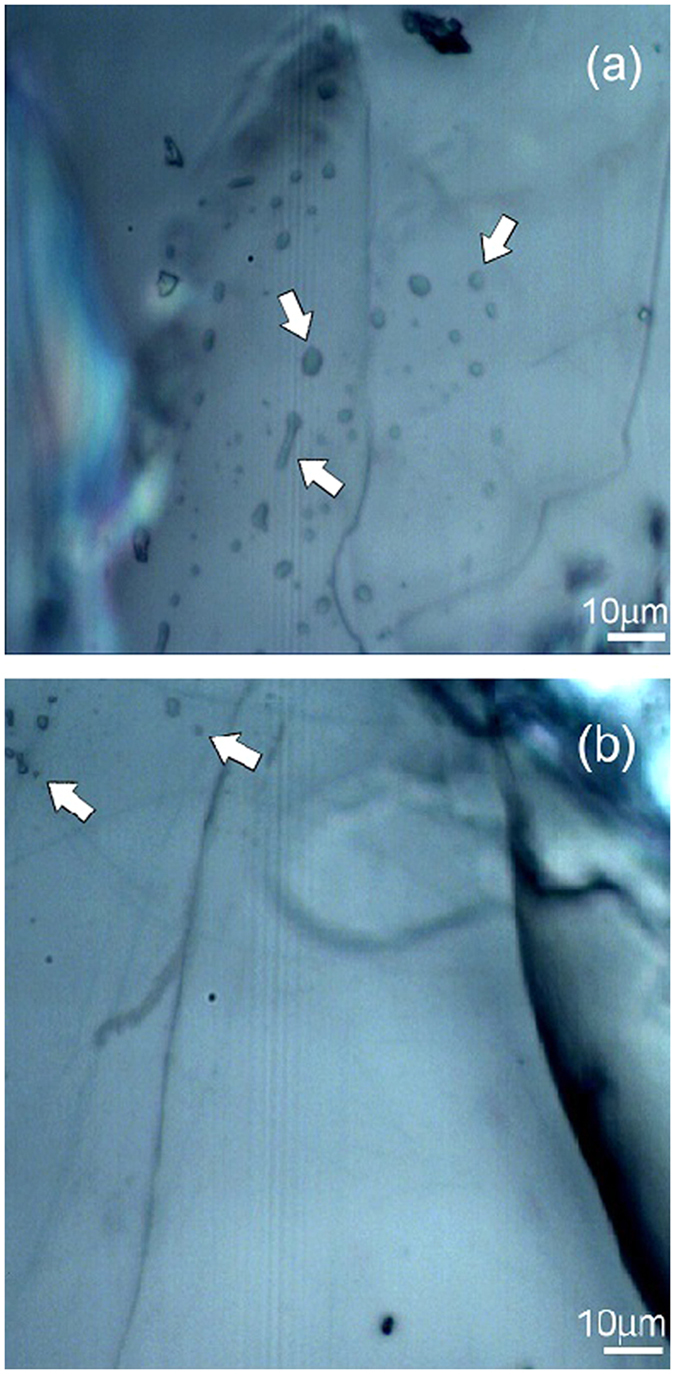
Optical images of a portion of BU1-7 (**a**) and BU1-15 (**b**) phlogopite samples where bubble-type inclusions were observed (see white arrows).

In order to identify the position of the nano-protrusions detected by atomic force microscopy for subsequent confocal and Raman analysis, digital optical images of the phlogopite crystals together with nanolithographic markers were recorded. It was found that the nano-protrusions have a correspondence with micro- and submicro-inclusions visible as bubbles by confocal microscopy (see white arrows in Fig. 5). Figure 5 shows, as a comparative example, two optical images of a 130 *μ*m × 130 *μ*m area of the BU1-7 (a) and BU1–15(b) phlogopite samples, where several pseudo-circular, elliptical and elongated bubbles are visible. Areas with a bubbles concentration of about 1.3 × 10^4^ bubbles/mm^2^ were observed in the BU1–7 sample, whereas areas with a concentration of about 8.7 × 10^2^ bubbles/mm^2^ in the BU1–15 sample. Bubbles were observed through the entire thickness of the sample.

#### The hosting phlogopite bubbles

**Figure 6:**
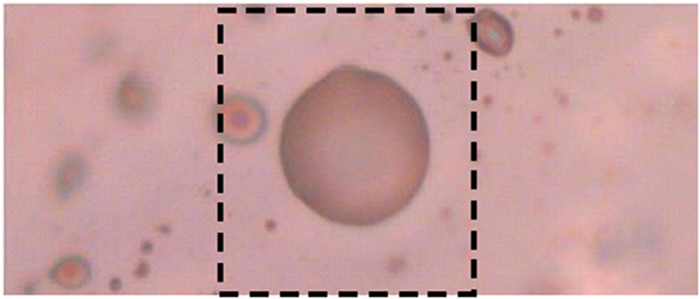
Optical image of the study area; the central bubble has a diameter of about 10 *μ*m.

A detailed FTIR study concerning the OH-stretching region and more generally the crystal chemistry of Bunyaruguru micas was done by Scordari and coworkers^11^. In the present paper, the identification of the material within the inclusions was addressed by Raman spectroscopy. Figure 6 shows a confocal optical image of the study area obtained with the camera of the Raman microscope. Several bubbles of different dimension are present; the one selected for the spectroscopic analysis is indicated by the broken-line box. The bubble has an almost perfect spherical shape, with a diameter of 10 *μ*m.

**Figure 7:**
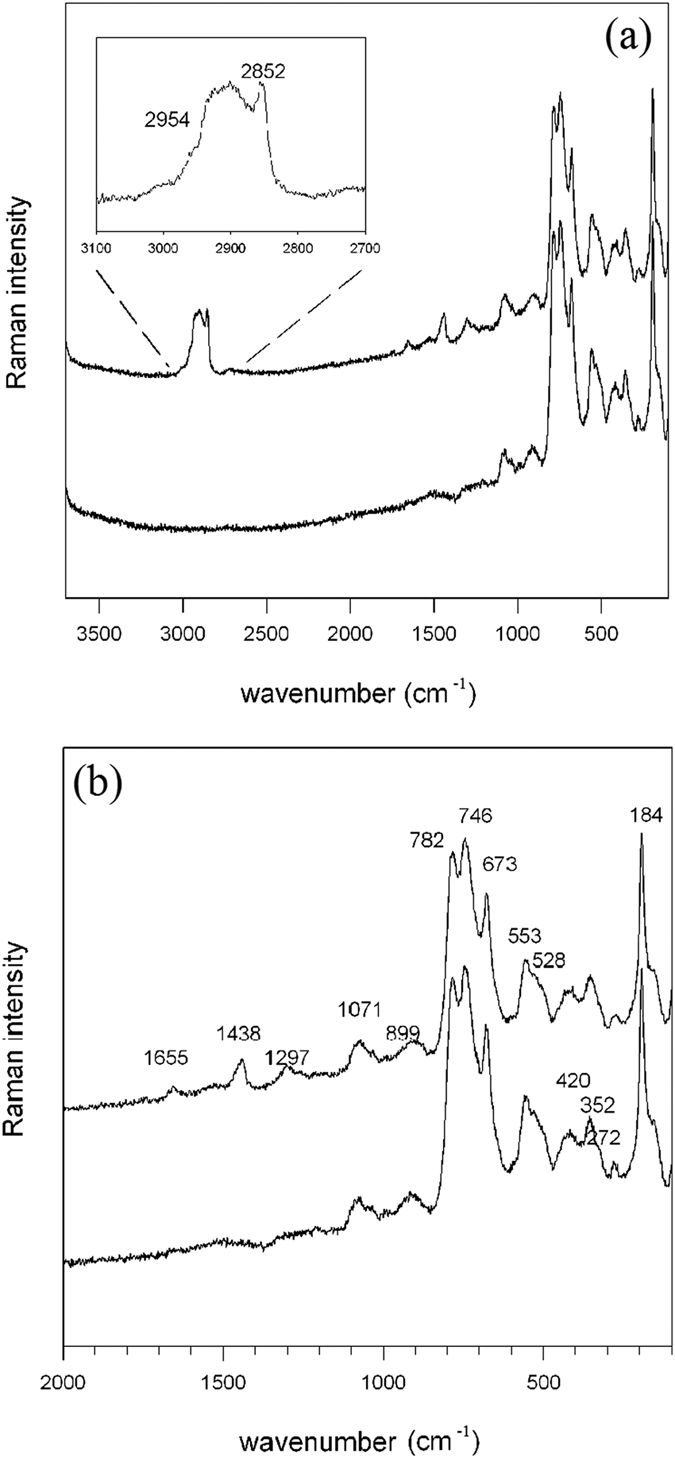
(**a**) Comparison of the single-spot Raman spectra collected outside (bottom) and inside (top) the bubble in Fig. 6. The enlargement of the 3100-2700 cm^−1^ range is given in the box. (**b**) Enlargement of the 2000–100 cm^−1^ range of the spectra showed in (**a**).

Figure 7a displays two selected single-spot Raman spectra collected outside and inside the bubble, respectively. The spectrum of the host mica (bottom pattern in Fig. 7) shows two weak and relatively broad bands at 1071 and 899 cm^−1^, a triplet on intense peaks at 782, 746 and 673 cm^−1^, and a series of lower-frequency bands at 553, 528, 420 and 352 cm^−1^ (Fig. 7b), respectively. An intense and very sharp peak is finally present at 184 cm^−1^ (Fig. 7b). Following the literature on these compounds^12^,^13^,^14^, the weak bands in the 1100–800 cm^−1^ range can be assigned to the antisymmetric internal modes of the Si-O layer, the strongest bands in the 600–800 cm^−1^ range to the symmetric Si-O-Si modes, while the region at wavenumbers <600 cm^−1^ is dominated by Si-O deformation modes, internal MO_6_ vibrations and M-OH librations.

According to the crystal-chemical data^5^,^6^, the studied sample can be classified as Ti-rich phlogopite; octahedral constituents other than Mg and Ti, i.e. Al, Fe^3+^, Cr and Ni (see above), are present in very low amounts. The Fe number (Fe# = Fe^2+^/(Mg + Fe^2+^) is thus very low (Fe# = 0.08) for both polytypes. Raman data for Mg-Fe trioctahedral micas along the phlogopite - annite system (biotites) have been published by several authors^13^,^14^,^15^,^16^. A comprehensive discussion for the interpretation of the Raman spectra of layer silicates, as a function of their structural variants and chemistry, has been recently provided by Wang and co-workers^17^. According to the latter authors, end-member phlogopite shows a very intense peak at 682 cm^−1^; for increasing Fe to Mg substitution at the octahedral sheet (Fe# 0.17) this peak splits into a well-resolved triplet of bands whose positions are a linear function of the Fe number^17^. Polarized measurements^15^ indeed show that the intensity of these peaks is strongly dependent on the orientation of the crystal with respect to the laser beam. Biotite from Horni Slavkov^15^ (Czech Republic) having a Fe# = 0.37 shows a triplet of bands at 773, 725 and 676 cm^−1^, in line with the spectra given by Wang *et al*.^17^. The sample studied here, on the contrary, although characterized by a very low Fe# shows the three-band pattern in the 800–600 cm^−1^ range (Fig. 7b) typical of biotites with higher Fe contents; this suggests that the relationship between the octahedral composition and the multiplicity of the Raman bands in the 800–600 cm^−1^ range is probably not a simple function of the Mg/Fe^2+^ ratio, but all cations substituting for Mg [in our case, the fairly high Ti resulting in a [(Fe_*tot*_ + Ti)/(Mg + Fe_*tot*_ + Ti) = 0.19] could have an effect.

#### The organic content of the bubbles

**Figure 8:**
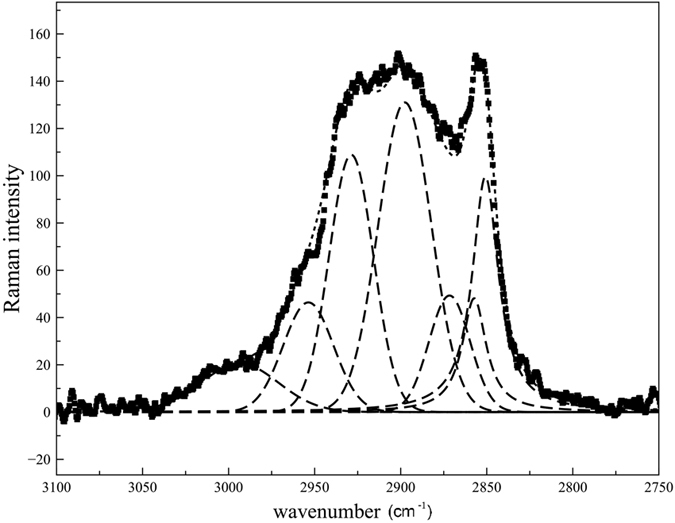
Spectral resolution between 3000 and 2800 cm^−1^.

The spectrum collected inside the bubble (top pattern in Fig. 7a and b) is identical in the lower wavenumber range (<1100 cm^−1^), but displays some new bands at higher wavenumbers: one broad absorption with manifold components in the range 3000–2800 cm^−1^ (Fig. 7a) and three distinct features at 1655, 1438 and 1297 cm^−1^ (Fig. 7b). One additional weak component is probably present on the lower-frequency side of the 1297 cm^−1^ peak, at 1260 cm^−1^ (7b). The multicomponent band in the 3000–2800 cm^−1^ range, magnified in the inner box of Fig. 7a, consists of an intense peak with a maximum at 2852 cm^−1^, a broad feature centred around 2900 cm^−1^ with an evident shoulder on the higher frequency side, at 2954 cm^−1^ and a weaker component at 3000 cm^−1^. The spectral region between 3000 and 2800 cm^−1^ is typical of C-H stretching vibrations of aliphatic hydrocarbons, namely C-H of methyl (bands at ca. 2960 and 2870 cm^−1^ for asymmetric and symmetric stretching vibrations, respectively) and methylene groups (ca. 2900 and 2850 cm^−1^ for asymmetric and symmetric stretching, respectively). In order to solve the different components embedded in the 3000–2800 cm^−1^ band, a mathematical treatment was carried out, by resolving the broad absorption through interactive optimization followed by least square refinement; the background was treated as linear and all peaks were modelled as Gaussians except the higher frequency peak at 2852 cm^−1^ that could be treated as Lorentzian. The minimum number of peaks needed to describe the experimental envelope was introduced in the refinement. The final result is given in Fig. 8; seven peaks can be fitted to the experimental envelope at 2993 (weak), 2954 (medium), 2929 (strong), 2898 (strong), 2872(medium), 2857 (medium) and 2850 cm^−1^ (strong), respectively. Specifically the bands at 2872 and 2954 cm^−1^ (both of medium intensity) can be attributed respectively to the symmetric and asymmetric C-H stretching vibrations of methyl (-CH_3_) groups whereas those at 2850 and 2898 cm^−1^ (both of strong intensity) to the C-H stretching vibrations of methylene (-CH_2_-) groups. This assignment is confirmed by the evident bands at 1438 and 1297 cm^−1^ attributed to the methylene (-CH_2_-) scissoring and twisting modes, respectively. Interestingly, the bands at 2857 and 2929 cm^−1^ (Fig. 7b) can be reasonably assigned respectively to the symmetric and asymmetric C-H stretching vibrations of -CH_2_- groups of C_6_-C_8_ cycloalkanes^1^. This is further confirmed by the small feature at 1260 cm^−1^ attributable to the -CH_2_- twisting vibration of cycloalkanes. These results are a clear indication of the presence of a mixture of alkanes and cycloalkanes compounds. In our case, the higher intensity of the bands of methylene groups compared to those of methyl groups could suggest that the hydrocarbon chains are quite long^18^,^19^. Indeed, the intensity ratio between -CH_2_- and -CH_3_ has been often related to the number of methylene groups and, therefore, to the length of the hydrocarbon chain^18^. It has been also reported that, when the hydrocarbon chains are in the liquid state, the dominant feature in the C-H stretching region of the Raman spectra is the peak at about 2850 cm^−1^, whereas when the hydrocarbons are in the solid state a peak at 2880–2890 cm^−1^ is the predominant one^20^,^21^. The weak feature at 3000 cm^−1^ (box in Fig. 7a) could be attributed to the C-H stretching vibration of =C-H groups of unsaturated compounds. This attribution seems supported by a band at 1655 cm^−1^ that could be assigned to the C=C double bond stretching vibration, thus pointing to the presence of small amounts of unsaturated aliphatic hydrocarbons. Similar band positions and assignment were proposed for fluid inclusions in a late syn-deformational sparitic calcite vein of the Hoh accretionary complex, Olympic Peninsula^1^. It must be however underlined that the presence of unsaturated hydrocarbons is extremely rare in natural occurring oils^22^. Therefore, according to Orange and coworkers^1^, it is possible to propose an alternative attribution of the 3000 and 1655 cm^−1^ bands, namely to the O-H (3000 cm^−1^) and C=O (1655 cm^−1^) stretching vibrations of carboxylic acids.

Finally, the presence of aromatic groups can be ruled out because no typical features due to aromatic compounds are visible in the spectrum (namely aromatic C=C stretching at about 1600 cm^−1^ and aromatic C-H stretching at wavenumber greater than 3000 cm^−1^).

Summarizing, the above considerations reasonably suggest that the matter found inside the bubbles consists of a liquid fraction made essentially of aliphatic hydrocarbons (both alkanes and cycloalkanes) with a quite long carbon chain. The small amount of oxygenated compounds, such as carboxylic acids, is noteworthy, although the presence of unsaturated hydrocarbons could not be excluded.

**Figure 9:**
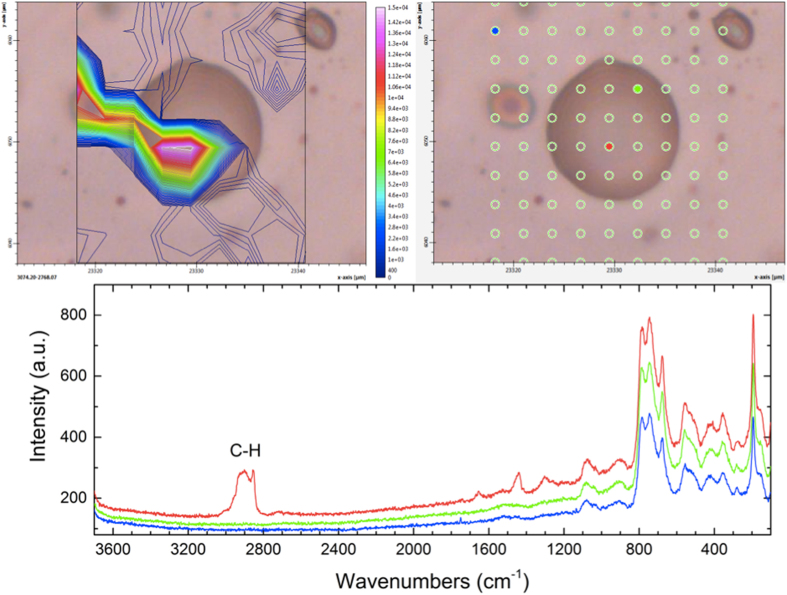
Top right: analytical grid across the studied bubble. The colored spots correspond to the spectra given below. Bottom: Raman spectra collected at the colored spots in the image above. The spectrum in red (red point within the bubble) clearly shows the additional bands assigned to C-H groups (see text). Top left: resulting Raman image obtained by integrating the absorbance in the 3000–2800 cm^−1^ range (indicated in the figure) for all points.

Several single-spot analyses were carried out and all gave the same result. Thus, in order to characterize the distribution of the hydrocarbons within the bubble, we collected a grid of single spots across a selected area, moving the microscope stage with a 2 *μ*m step (Fig. 9).The intensity in the 3000–2800 cm^−1^ range was integrated to finally obtain an image of the distribution of the hydrocarbons across the study area. Figure 9 (top left) clearly shows that these are distributed exclusively within the bubble, and in particular in the lower part. A similar pattern was obtained within a second protrusion from a different area. The Raman imaging thus indicates that the bubbles observed on the surface of the phlogopite flakes are filled by organic matter composed mainly of aliphatic hydrocarbons with relatively long chains; the distribution of these molecules on one side only of the bubble is consistent with their liquid nature, as inferred on the basis of the Raman spectra (see above).

## Conclusions

AFM analysis showed that both phlogopite polytypes from Kasenyi kamafugitic rocks (SW Uganda) have similar surface potential, and allowed detecting and characterizing in detail surface nano-protrusions, particularly abundant in the 1*M* polytype, with heights varying from some nanometers to tens of nanometers and a lateral extension of the order of hundreds of nanometers to several micrometers. An aspect ratio (maximum height/maximum width) from 0.01 to 0.09 was generally observed. Correlated AFM and confocal microscopy investigations were able to associate the surface nano-protrusions to the presence of sub-surface bubbles; Raman spectroscopy indicated that these micro- and sub-micro sized bubbles were filled by fluid aliphatic hydrocarbons, although a small amount of oxygenated compounds is present. In addition to that, the presence of unsaturated hydrocarbons could not be excluded.

The amount and location of carbon in Earth is a question of paramount importance in geochemistry, and this is particularly true for deep carbon storage systems and their fate in the dynamic of the Earth^23^. This work allowed to observe directly, for the first time that phlogopite, a common rock-forming mineral, may act as a possible reservoir for hydrocarbons. The question whether the inclusions are primary, i.e. syngenetic with the rock formation, or secondary, i.e. related to hydrothermal phenomena, is not trivial. The studied phlogopite samples are from a mantle-derived nodule^5^, pointing to a peculiar occurrence of deep origin hydrocarbons. Both field evidence and experimental work suggest a very fast magma ascent for carbonatite-kamafugite products, were the propellant is provided by abundant juvenile CO_2_-rich volatile^24^. In the presence of water, a reaction between CO_2_ and H/H_2_O to form hydrocarbon molecules is feasible^25^,^26^, although at P/T conditions different to those of a deep-seated system. The pervasive nature of the protrusions, any lack of evidence of secondary alteration both in the host rock and in the studied phlogopite crystals^5^,^24^ suggest an early genesis for the hydrocarbon-filled bubbles. However, a definitive assessment could be provided only by C and H isotopic data^27^ which are unavailable at the moment, due to the difficulty to obtain this information on such small samples.

The important implication of this work is that Nature offers unexpected hydrocarbon reservoirs that still need to be characterized.

## Materials and Methods

### Occurrence

The phlogopite single crystals analyzed here were hand picked under the binocular microscope from the crashed rock sample BU1^24^,^28^. The BU1 rock sample occurs within the ugandan kamafugite, collected at Bunyampaka maar (Kasenyi field), and is a melilitite bomb described as a lapilli tuffisite by Stoppa *et al*.^24^. In this rock the association of kamafugite and carbonatite is predominant. At present this association has been observed only in four areas: Katwe-Kikorongo and Bunyuaruguru in Uganda, IUP localities in Central Italy, Mata da Corda in Brazil and Qinling in Gansu, China (Stoppa *et al*.^24^).

In the ugandan tuffisite subhedral phlogopite crystals (20%) are associated to olivine (40%), melilite (25%) and spinel (15%). The ugandan kamafugite, as well as those from West Quinling (China) and Brazil, are typically enriched in incompatible elements with high LREEs, low HREEs and thus elevated LREEs/HREEs ratios, abundance of Rb, K, P and Ti and negative Rb, K, P and Ti anomalies^28^. In addition, the ugandan kamafugites are associated with carbonatitic rocks, similarly to the kamafugites from Italy^24^. The petrogenetic relationships between kamafugite and carbonatite have not been definitively assessed. Three different hypothesis have been reported^29^: (a) carbonatite derived by the crystallization of carbonate minerals from a silicate parental magma; (b) carbonatite and kamafugite derived by different parental magma; (c) carbonatite and kamafugite solidified by two immiscible liquids (a carbonatite magma and a silicate magma) from a common CO_2_-rich silicate parental magma. On the basis of geochemical, stratigraphic and petrographic features, the latter hypothesis was recently confirmed to describe the origin of the China kamafugite-carbonatite association^28^. The authors also argued that the process of melt immiscibility likely occurred during the magma ascent from depth as a result of the decrease of the CO_2_ solubility in the host melt, thus a melt with an original homogeneous composition segregated into two different magmas. The hypothesis indeed was found to be effective in explaining the petrogenesis of all the worldwide carbonatite-kamafugite associations.

### Atomic force and confocal microscopy

Investigation of the surface properties of the phlogopite crystals was done by using a Multimode Atomic Force Microscope (Digital Instruments, Santa Barbara, California) interfaced with a Nanonis control system (Nanonis – SPECS Zurich GmbH, Zurich, Switzerland) and equipped with two oscillation controller modules (with digitally integrated PLL/lock-in). The precise control of the relative tip-sample position was achieved by an nPoint closed loop MultiMode scanner, 100 *μ*m scan range, with a C-300 DSP closed-loop controller (nPoint, Inc., Madison, WI, USA). The analyses were performed in both contact and intermittent contact mode.

Bruker MPP-11100 silicon rectangular probes (Bruker Co., Camarillo, CA, USA) with nominal force constant, k, 40 N · m^−1^, nominal resonant frequency 300 kHz and nominal tip radius of curvature 10 nm were used for the morphological characterization in intermittent contact mode. Bruker NP type silicon nitride triangular probes with nominal force constant, k, between 0.06 N · m^−1^ and 0.58 N · m^−1^ and nominal tip radius of curvature 20 nm were used in contact mode. Furthermore, Kelvin probe mode^9^ was employed to investigate surface electrostatic potential variations by means of Nanoworld EFM type and Arrow-EFM type Pt-Ir coated silicon rectangular probes (Nanoworld AG, Neuchâtel, Switzerland). Both probes have nominal force constant, k, 2.8 N · m^−1^, nominal resonant frequency 75 kHz and nominal tip radius of curvature 25 nm. All the AFM observations were conducted in air, at atmospheric pressure, RT between 20 °C and 25 °C, and RH between 35% and 45%.

Optical images of the areas investigated by AFM, marked with specific nanolithographic signs, were recorded in order to identify the position of the nano-protrusions (see section Results and Discussion) for subsequent confocal and Raman analysis. The optical images were collected by means of a Nikon trinocular optical microscope equipped with a CMOS digital camera coupled to the AFM system. An Olympus BX41 confocal microscope with 10x, 50x and 100x objectives and motorized XYZ stage was then used to optically investigate the nano-protrusions previously detected by AFM. We discovered that the nano-protrusions were related to sub-surface bubbles or inclusions.

### Raman microscopy

The nature of the bubble-filling material was assessed using Raman spectroscopy. The phlogopite samples were positioned onto a Raman microscope stage fully automated for high-precision, computer controlled, x-y movements. Raman spectra were collected at beamline B22, Diamond Light Source (DLS), Oxford (UK), using a confocal Bruker Senterra Raman microscope, equipped with a multichannel charge-coupled device (CCD) detector. Raman spectra were excited with the 532 nm laser, integration time 10 sec per scan, 2 scan per point, with a 50x objective. The laser power was kept low enough (12 mW) to avoid laser-induced degradation of the analyzed compounds. Three different gratings were employed to optimize the scattered signal. After the analyses, the sample was inspected optically for any laser damage, and none was observed. The wavenumber accuracy was 0.5 cm^−1^, and the spectral resolution was better than 1 cm^−1^. The measuring grid was set at step of 2 *μ*m, and the final image was obtained by integrating the Raman intensity in the 3000–2800 cm^−1^ range.

## Additional Information

**How to cite this article**: Moro, D. *et al*. Hydrocarbons in phlogopite from Kasenyi kamafugitic rocks (SW Uganda): cross-correlated AFM, confocal microscopy and Raman imaging. *Sci. Rep.* **7**, 40663; doi: 10.1038/srep40663 (2017).

**Publisher's note:** Springer Nature remains neutral with regard to jurisdictional claims in published maps and institutional affiliations.
